# The level of household satisfaction with community-based health insurance and associated factors in Southern Ethiopia

**DOI:** 10.3389/fpubh.2023.1165441

**Published:** 2023-06-30

**Authors:** Kebebush Zepre

**Affiliations:** Department of Public Health, College of Medicine and Health Science, Wolkite University, Wolkite, Ethiopia

**Keywords:** level of satisfaction, CBHI, associated factors, community, Southern Ethiopia

## Abstract

**Background:**

Community-based health insurance (CBHI) is a program intended to prevent financial hardship brought on by the cost of medical care. All of Ethiopia’s regions are implementing it; however, it has not yet been researched how the program is being received by the local population. This study’s objective is to determine how satisfied Southern Ethiopian households are with community-based health insurance programs and connected variables.

**Methods:**

A community-based cross-sectional study was conducted from April to May 2021. Information was gathered from 528 households (HHs) selected at random in the Gurage Zone of Southern Ethiopia using a questionnaire. Bivariate and multivariate logistic regression, as well as descriptive statistics, were applied. *p* values less than 0.05 was used as a cutoff point for identifying the self-determining factors.

**Results:**

The adjusted odds ratio (AOR) for HHs with the poorest wealth status was 2.40 (95% confidence interval:1.14–4.90); for HHs with a good knowledge of the CBHI, it was 1.81 (95% CI: 1.87–3.40); and for households with illness in the past 3  months, it was 5.22 (95% CI: 2.91–9.34). Recurrent visits to the facility (AOR:5.04, 95% CI:1.18-23.44), a Model household in rural health extension program (AOR:3.21, 95% CI:1.76-5.85), being enrolled in the scheme for three years or less (AOR:0.55, 95% CI: 0.30-0.95), and having faith in the leadership of the governing board (AOR:10.53, 95% CI:4.690-23.54) and the availability of the prescribed medication (AOR:14.64, 95% CI:5.37-39.84) were the significant influencing factors.

**Conclusion:**

This study revealed several variables that affected HHs’ satisfaction with CBHI. We strongly advise all responsible parties to focus on increasing HH knowledge of the CBHI scheme, supporting HHs to serve as role models for rural health extension packages, and completing the CBHI pledged package to improve HHs’ satisfaction with the CBHI scheme, which may then play a role in the sustainability of CBHI.

## Introduction

An important component of how the health system operates is how health care is financed. The biggest challenge for the international development community is how to pay for and deliver health care for the rural poor and unemployed in undeveloped countries (1, 2). The bulk of the impoverished in these countries cannot afford or even access health care (3). Many people find it challenging to pay directly for medical services, and millions of people experience hardship as a result of having to do so (4). As a consequence, they continue to have health issues despite the fact that they may be prevented or treated with the right care (5).

The purpose of community-based health insurance (CBHI) is not to make a profit. In the CBHI program, participants pay a modest annual contribution to a pooled fund that helps them with emergency expenses during sickness. It primarily benefits the underprivileged and enrollment is optional. It has the benefit of narrowing the equity gap and lowering out-of-pocket expenses, raising knowledge of the value of insurance, boosting participant confidence through a system of community control, and increasing the use of healthcare services (6). The major roadblocks to using and accessing health services are financial (7).

The sustainable development goals call for international cooperation to achieve universal health care by 2030. In spite of this, at least 50% of citizens worldwide still do not have access to basic health care. As a result of having to spend a sizable portion of their household budgets on medical bills, a lot of people are being forced into poverty; almost 150 million people worldwide experience financial disaster each year (8–11).

In Ethiopia, easily curable communicable illnesses continue to be a serious public health issue (12). However, rural communities have low levels of health-seeking behavior and modern healthcare access (13, 14). The explanation for the lack of access to basic health care is direct out-of-pocket payment, which can cause psychological and economic problems for families (15, 16). Therefore, to overcome this financial hardship, the Ethiopian government has started two types of health insurance schemes. The first is community-based health insurance (CBHI) and the second is social health insurance (SHI). CBHI is a step toward averting the economic adversity linked with paying for health care (17). In Ethiopia, CBHI schemes have been established since 2010 to alleviate the financial challenges brought on by out-of-pocket expenses. The initiative is growing and there is an intention that it will cover 85% of Ethiopians who work in the informal sectors. It is planned that the SHI scheme will cover 10.46% of the population who are engaged in the formal sectors, and it is currently in the process of being implemented (18).

By adopting the CBHI plan in rural regions, the Ethiopian government is attempting to bridge the current gap between society’s need for healthcare and the budgetary limitations of the healthcare industry (19). Presently, 161 districts are using the CBHI plan, and a recent review revealed improvements in health service consumption in those districts (18). However, approximately 23% of enrollees drop out of CBHI due to dissatisfaction, according to recent reports from the Ethiopia health insurance agency (EHIA); CBHI coverage is 28%, which is low compared with the HSTP target of 80%. Additionally, the southern region has seen a 10% increase in its dropout rate. This puts the EHIA’s goal of ensuring complete coverage with CBHI by 2025 in serious doubt (20, 21).

HHs’ satisfaction with the CBHI scheme is the pleasant feeling that users get when they use health care services through the CBHI system in relation to the trustworthiness of the CBHI committee and satisfaction with drug availability, the processing of insurance cards, member waiting times for service, annual contribution payment times, the information provided, and the CBHI packages (6). Studies conducted previously in Ethiopia and other countries on HHs’ satisfaction with CBHI revealed the following results: 54.7% in Sheko district Ethiopia (22); 91.4% in Ethiopia (23); and 55.9% in Istanbul (24).

According to several studies, most of socio-demographic factors have an impact on enrollees’ satisfaction with their health insurance (22–25). The satisfaction of enrollees with CBHI is also influenced by characteristics relating to health services. The enrollee’s impression of the quality of laboratory services, the friendliness of the healthcare professional, the speed of processing, and the ease of drug use are all closely linked with their satisfaction with CBHI (22–26). Additionally, research from developing nations has demonstrated that an enrollee’s understanding of the benefits of the health insurance program affects their happiness with the program (22–25, 27).

In addition, HHs’ satisfaction is affected by variables related to HHs’ CBHI utilization (23). Since 2011 EC, the CBHI program has been ramped up in this study area. However, the level of satisfaction and correlated factors have not been studied. Only a few such investigations have been carried out in other parts of Ethiopia, as far as we are aware (22, 23). Thus, this study was intended to assess the level of HHs’ satisfaction with CBHI and associated factors.

## Methods

### Study design and setting

This cross-sectional study was carried out at a community level from April to May 2021 in selected CBHI-implementing districts in the Gurage Zone in Southern Ethiopia. The Gurage Zone is one of the administrative zones of the SNNPR. It is sited 155 km southwest of Addis Ababa. In 2019, the population of the Gurage Zone was estimated to be 1.8 million, of which approximately 90% reside in rural areas. The Gurage Zone consists of 21 districts (16 rural and 5 urban). Before, 2012 EC, only 10 of these rural districts were using CBHI. There are currently 24 medium-sized and 83 primary private clinics, 67 health facilities, 412 rural health posts, and six public hospitals (28).

### Populations

All CBHI users in the Gurage Zone’s rural CBHI implementation areas were considered as the source population. CBHI scheme participants who used health care services from a contractual facility in the last 3 months and were chosen for an interview were considered as the study population; the selected household heads are the study unit.

### Inclusion and exclusion criteria

Household heads who were enrolled in the CBHI scheme and who used health care services from a contractual facility in the last 3 months were included. HHHs who were critically ill and incapable of responding were excluded.

### Sample size determination

To calculate the sample size, we used a single population formula (Zα/2)^2^ p(1-p)/d2 assuming *p* = 91.38% from a previous study (23), a confidence level of 95% and a margin of error of 0.03, a design effect of 1.5, and a non-response rate of 10%. Finally, a sample size of 528 was calculated.

### Sampling methods

A three-stage random sampling procedure was used. In the initial phase, 30% of the CBHI implementing districts were chosen via a lottery. Similarly, 30% of the kebeles under the designated districts were picked. Lastly, enrollees were selected from the registration using ID numbers with the assistance of health extension workers. Following that, each kebele received a proportionate share of the sample size. Finally, systemic sampling techniques were used to choose the study participants. Approximately three visits were made to those not present at the time of data collection; if not available at the third visit, the next household was chosen.

### Data collection and quality assurance

A prearranged survey form adapted from a variety of previous publications (23, 24) was used. It was prepared in English and transformed to Amharic and back to English to keep its uniformity. The Amharic version was used to collect the data. The tool was used to collect data on socio-demographics and economics, knowledge of CBHI, and CBHI scheme-related variables and others (23). The data collection and supervision were undertaken by trained nurses and senior public health professionals, respectively. To ensure data quality, 2 days of training were provided to data collectors and supervisors on the objectives, methods, and procedures of the data collection process. A pretest of the tool was made 1 week before the main data collection period with 5% of the total sample to check the consistency, clarity, and sequence of the questions, and also to make them familiar with the tool. Supervisors and principal investigators checked the completeness, accuracy, and consistency on a daily basis, after which basic amendments were made. Data were entered using Epi-data 3.1 before exportation to SPSS for analysis.

### Data analysis

Epi-data version 3.1 and SPSS version 21 were used for data entry and analysis, respectively. Descriptive statistics were obtained and presented as text, tables, and figures. HHs’ economic status was categorized into five wealth quintiles using principal component analysis (PCA) and subsequently recategorized in three. The association between factor and outcome variables was determined using binary logistic regression. Hosmer and Lemeshow statistics were used to test the fitness of the model and a good fit was found (*p* = 0.42). To assure the reliability of the tools used we considered Cronbach’s alpha coefficient. To control all potential confounders, we incorporated all variables with *p* < 0.25 in the bivariate analysis to the multivariate analysis. Numerical and graphical methods were used to test normality. The data point was close to the oblique line in a Q-Q plot test that shows the data were in a normal distribution. In the histogram, a bell-curve shape indicated the data was from a normal population. Variables with a VIF above 10 and a significant correlation in a multicollinearity test were excluded from the model. There was no significant effect alteration among factors in the last model, as shown in the multicollinearity and interaction effect analysis. The adjusted odds ratio with a 95% CI and a *p* < 0.05 was used to identify independent factors of HHs’ satisfaction with the CBHI scheme.

## Measurements

### Knowledge of the CBHI scheme

Eight knowledge assessment questions were used as follows: (i) CBHI is prepayment for health care and sharing financial risk among members, the range of benefit package (what type of health care service is allowed to use free of fee or not); (ii) service is not permitted from a private health institution; (iii) transportation expense is not included in the package; (iv) CBHI package encompassed inpatient and outpatient care; (v) cosmetic medical care is not incorporated; (vi) the amount and timing of premium payment; (vii) CBHI does not mean paying tax to the government; and (viii) CBHI does not mean free health delivery by the government. Then, households who scored ≥ median of the knowledge assessment questionnaire were considered as having good knowledge; those scoring less than the median were considered as having poor knowledge (23, 29).

### Household wealth status

HH wealth status is a measure of household living status that was created from HH asset data and consists of a variety of factors. It was updated from EDHS 2016 (30) to account for local agricultural goods generated in rural and local home contexts. Information about the type of floor, roof, and wall, the water supply, the toilet, radio, bicycle, and motorcycle ownership, the quantity of grain (gathered in the most recent production year), the number of livestock, and the ownership of farmland were used to measure it. HHs’ wealth was divided into quintiles after a principal component analysis (PCA) with SPSS. The quintiles were numbered from lowest (Q1) to highest (Q5) in order of increasing wealth (highest).

### Respondent’s overall satisfaction with CBHI

HHs’ satisfaction with CBHI was assessed by means of nine satisfaction-related items, as follows: (i) CBHI committee trustworthiness, (ii) satisfaction with drug availability, (iii) satisfaction with insurance card processing, (iv) satisfaction with waiting time for members for services, (v) satisfaction with annual contribution payment time, (vi) satisfaction with the information provided, (vii) satisfaction with the CBHI packages, (viii) interest in remaining enrolled, and (ix) motivating others to enroll in CBHI. The total score of each respondent was computed and scored a minimum of 18 and a maximum of 45 points. Consequently, if a HH’s response to the satisfaction questions was the median score or above, it was classified as satisfied; if the response was below the median, then it was classified as not satisfied.

### Ethics-related matters

The Institutional Review Board (IRB) of Wolkite University’s College of Medicine and Health Science permitted the study to proceed. The Gurage Zone health department and the relevant districts provided a letter of permission. By assuring respondents that their names and other identifiable information would not be written on the questionnaire, the respondents’ privacy was safeguarded. Respondents received clear explanations of the study’s objectives and the specifics of the consent procedure in the language of their choice. Finally, prior to data collection, written informed consent was obtained from each respondent. All respondents who were willing to participate signed the paper (even those who were illiterate added their signature by fingerprint). Respondents were informed that they had the full right not to participate and to discontinue at any time if they did not feel at ease.

## Results

### Socio-demographic characteristics of the participants

Five hundred and thirteen (513) household heads participated, resulting in a 97.1% response rate. Of these, 361 (70.4%) were men. The average age of the participants was 47.2 ± 11 SD years, and 89.9% of the respondents were married. The typical family size was six people, and more than half (55%) of the homes had more than 5 family members. A total of 221 (43.1%) individuals were illiterate. There was a virtually equal distribution across wealth categories (19.9–20.3%) (Table 1).

**Table 1:** Socio-demographic and economic characteristics of respondents of the level of households satisfaction to CBHI scheme and associated factors, in southern Ethiopia, 2021 (*n*=513).

Characteristic	Category	Count	(%)
Sex	Male	361	70.4%
	Female	152	29.6%
Age (mean age 47.2 ± 11 SD)	18–34	49	9.4%
	35–64	426	83.0%
	≥65	39	7.6%
Religion	Orthodox	281	54.8%
	Muslim	177	34.5%
	Others	55	10.7%
Marital status	Married	451	87.9%
	Widowed	52	10.1%
	Not married	10	2%
Level of education	Cannot read and write	221	43.1%
	Read and write only	78	15.2%
	Primary education (1–8)	171	33.3%
	Secondary and above	43	8.4%
Wealth in quintiles	Lowest (1st) wealth quintile	102	19.8%
	2nd wealth quintile	103	20.1%
	Middle wealth quintile	104	20.3%
	4th wealth quintile	102	19.9%
	Highest (5th) wealth quintile	102	19.9%
Family size	≤5	282	55%
	>5	231	45%

### Respondent’s knowledge of the CBHI scheme

Eight items were used to measure HHs’ knowledge of the CBHI scheme. Respondents’ knowledge of CBHI was assessed using eight items and were classified as having good knowledge if they scored median or above. Otherwise, they were classified as having poor knowledge. Accordingly, only 374 (72.9%) of the participants had good knowledge.

### Respondent’s experience of CBHI

Approximately 287 (55.9%) participants remained enrolled for three or more years. Approximately 406 (79.1%) HHs perceived the annual premiums as affordable. Only 14 (2.7%) participants reported that their premiums were covered by government. Approximately 402 (79.5%) participants reported that they trusted the CBHI committee. Approximately 61 (11.9%) participants reported that ordered drugs were not available. Approximately 64 (12.4%) particpants perceived that there is service provision partiality between members and non-members. Respondents also reported high annual premiums (58, 11.3%), narrow benefits packages (39, 7.6%), and long waiting times (53, 10.3%). Approximately 336 (65, 5%) respondents reported that they wanted to renew their CBHI membership year after year.

### Level of satisfaction with the CBHI scheme

**Figure 1:**
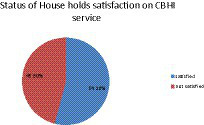
The satisfaction of respondents with the CBHI scheme and associated factors in Southern Ethiopia, 2021.

In this study, satisfaction levels were assessed using nine items. Among the study participants, approximately 278 (54.2%) respondents scored the median or above for the satisfaction questions and were considered as satisfied; the remaining 45.8% of the respondents were considered as not satisfied (Figure 1).

### Factors connected with respondents’ satisfaction with the CBHI scheme

In the bivariate binary logistic regression analysis, marital condition, family size, presence of children under 5 years of age in the HH, wealth status, exposure to health facilities, illness, trust in the CBHI board, drug availability, and the model status of HHs were significantly associated with respondent’s satisfaction with CBHI. Those variables that were significant (*p* ≤ 0.25) were chosen and tested for independence by multivariate analysis. A backward stepwise LR (likelihood ratio) logistic regression method was used. In the final model, eight variables were independently significant (*p* < 0.05).

Accordingly, the odds of being satisfied with the CBHI scheme among HHs belonging to the poorest economic status (1st and 2nd quintiles) were higher than those of the wealthiest households (AOR: 2.40, 95% CI: 1.14–4.90) (Table 2). Respondents’ knowledge of CBHI had a strong association with HHs’ satisfaction with the CBHI scheme. The likelihood of respondents being satisfied with CBHI was nearly 2 times higher among those with a good knowledge than those with a poor knowledge (AOR: 1.81, 95% CI: 1.87–3.40) (Table 2). Likewise, HH illness occurrence showed a strong association with respondent’s satisfaction with the CBHI scheme. HHs with ill health in the last 3 months were five times more likely to be satisfied (AOR: 5.22, 95% CI: 2.91–9.34) (Table 2). Similarly, the finding showed that frequent exposure of HH members to health facilities had a well-built association with HHs’ satisfaction with CBHI; HHs that frequently visited health facilities were five times more likely to be satisfied than those that visited less frequently (AOR: 5.04, 95% CI: 1.18–23.44) (Table 2).

**Table 2:** Factors associated with household level of satisfaction with the CBHI scheme adjusted for confounding variables, in Southern Ethiopia, 2021 (*n* = 513).

Variables	Category	Satisfied with CBHI		COR (95%C.I)	AOR (95%C.I)	*p* value
		Yes (%)	No (%)			
Household wealth status (in quintiles)	Lowest quintiles	136 (39.6)	69 (40.6)	0.76 (0.48–1.20)	2.40 (1.14–4.90)*	≤0.02
	Middle quintiles	60 (17.6)	44 (25.6)	1.43 (0.97–2.24)	1.68 (0.87–3.48)	
	Wealthiest quintiles	146 (42.8)	58 (33.8)	1:00	1:00	
Household knowledge about CBHI	Good	281 (82.2)	93 (54.4)	3.86 (2.56–5.81)	1.81 (1.87–3.40)*	≤0.050
	Poor	61 (17.8)	78 (45.6)	1:00	1:00	
Illness in the last 3 months	Yes	279 (81.6)	74 (43.3)	5.80 (3.81–8.72)*	5.22 (2.91–9.34)*	≤0.001
	No	63 (18.4)	97 (56.6)	1:00	1:00	
Exposure to health facility	>5 times/year	339 (99.1)	149 (87.1)	16.68 (4.91–56.6)*	5.04 (1.18–23.41)*	≤0.030
	≤5 times/year	3 (0.9)	22 (12.8)	1:00	1:00	
Graduated MHH	Yes	231 (67.5)	78 (45.6)	2.40 (1.7–3.60)*	3.21 (1.78–5.84)*	≤0.001
	No	111 (22.5)	93 (54.4)	1:00	1:00	
Duration of membership	>3 yrs	224 (65.5)	63 (36.8)	1:00	1:00	
	<=3 yrs	118 (34.5)	108 (63.2)	0.35 (0.21–0.45)*	0.55 (0.30–0.95)*	≤0.050
Trust in the CBHI committee	Yes	320 (93.6)	82 (48.0)	15.78 (9.3–26.73)*	10.54 (4.70–23.54)*	≤0.001
	No	22 (6.4)	89 (52.0)	1:00	1:00	
Ordered drug available	Yes	330 (96.5)	91 (65.0)	14.8 (7.55–29.01)*	14.61 (5.38–39.84)*	≤0.001
	No	12 (3.5)	49 (30.0)	1:00	1:00	

^*^Statistically significant.

On the other hand, being a model HH (graduated as a model in a rural health extension program) was strongly associated with satisfaction with the CBHI scheme; those respondents who graduated as a model were three times more likely to be satisfied with the CBHI program than those who did not graduate (AOR: 3.21, 95% CI: 1.76–5.84) (Table 2). Another finding from this study was that when a member remained enrolled in the program only for 3 years or less, it lessened the likelihood of being satisfied by 45% compared with those HHs that remained enrolled for >3 years. Likewise, trust in the CBHI governing bodies increased the satisfaction rate with the CBHI scheme 10 times compared with HHs that did not have trust (AOR: 10.54, 95% CI: 4.70–23.54) (Table 2).

In addition, this study showed that accessibility of the drug ordered in the agreed institution is strongly correlated with respondent’s satisfaction with CBHI; HHs that reported that the prescribed drug was available were 14 times more likely to be satisfied than HHs that reported that the ordered drug was not available (AOR: 14.63, 95% CI: 5.37–39.84) (Table 2).

## Discussion

In LICs, there is considerable disparity in health service delivery. CBHI may help the World Health Assembly in encouraging all countries to move toward universal health coverage (UHC) (31). Although the CBHI program is being implemented in some of the LICs, the documentation of its implementation is poor. At this juncture, we evaluated the satisfaction level of users in the CBHI scheme in the Gurage Zone of Southern Ethiopia. We found that participants were moderately satisfied with CBHI. This result is analogous with those of studies carried out in Ethiopia (22) and Istanbul (Turkey) (24). On the other hand, it is higher than the finding reported in Nigeria (29) but lower than that reported in a study in Ethiopia (23). This disparity might be due to measurement differences. For instance, in the study stated previously, 98.2% of respondents reported that the quality of health care service improved after the CBHI scheme was introduced in the district and they have been considered as satisfied (23).

In our study, none of the sociodemographic variables were significantly correlated with respondents’ satisfaction with CBHI in the final model except wealth status. A similar finding was reported in India (27). Nevertheless, respondents’ knowledge of the CBHI scheme, occurrence of illness among family members in the last 3 months, respondents’ frequency of health institution visits, length of enrollment in the scheme, HHs’ model status in the rural health extension program, and the accessibility of prescribed drugs in the contractual health institution were significantly associated with the satisfaction of respondents with the CBHI scheme.

Less rich HHs were more likely than wealthy HHs to report being satisfied. Research findings from Nigeria, Bangladesh, and Ethiopia have reported similar findings (23, 25, 26). Owing to their propensity for using the private sector, wealthy people may expect better than normal health services. However, the private sector may not be used by low-income HHs. Therefore, they may be easily content with the current service because they have lower expectations than rich enrollees.

Participants who had a better understanding of the CBHI scheme were more likely to be satisfied than those who had less of an understanding. Studies carried out in Ethiopia and Nigeria support this (22, 23, 25–27). It could be the case that enrollee satisfaction only increases if they are aware of CBHI benefit packages and how the health insurance program operates. It is possible that those who are more knowledgeable about CBHI benefits will gain more from them and be happier overall. Lack of understanding of the health insurance package has been shown to have a negative impact on how often LICs use their health insurance (31). The health insurance benefit packages should therefore be explained to all subscribers in the best way possible to ensure they are understood.

Drug availability was one of the aspects of health service provision that was substantially linked to satisfaction. More people were likely to report being satisfied than those who did not receive recommended medications or did not agree with them. This result is comparable with studies carried out in Ethiopia and Bangladesh (23, 25). This may have occurred because enrollees who did not receive prescribed medications in a government health institution were required to pay extra fees for private pharmacies, which may be the root cause of their dissatisfaction with the CBHI program. Another possible explanation for this is that contractual health facilities did not deliver the promised services, which undermines trust and leads to dissatisfaction.

Another significant finding from our study was that the longer enrollees remained in the scheme, the greater their level of satisfaction with it. This finding is similar to that from a study carried out in Nigeria (32) and may be because the longer they stay as a member, the more they may appreciate the benefit, and health service quality may improve through time thus increasing satisfaction. HHs reporting the occurrence of illness in a family member in the last 3 months were nearly five times more likely to be satisfied. This is analogous to the findings in a study undertaken in Bangladesh (25) and might be due to people who experience illness being more likely to remain in the scheme. Likewise, the finding showed that frequent exposure of HH members to health facilities has a strong association with satisfaction likelihood; HHs that frequently visit health facilities were five times more likely to be satisfied than their counterparts, perhaps because those who visit health facilities frequently may understand the financial catastrophe they face and be satisfied with the service they receive.

Another persuasive finding here is the model of respondents in a rural health extension package, with a model HH favorably associated with satisfaction with CBHI. The analysis illustrated that model HHs (graduated as a model in a rural health extension program) were strongly associated with satisfaction; those HHs that graduated as a model were three times more likely to be satisfied with CBHI than those that did not graduate, perhaps because model HHs might have a better understanding about CBHI benefit and have trust on the CBHI governing board, as the model HHs have been exposed to different local gathering and close relationship to the chain of administrative system than non-models.

This study also showed that HH distrust of the CBHI committee increased dissatisfaction rates with the CBHI approximately ten times compared with HHs that did trust the committee, which concurs with a study finding from Ethiopia and Cambodia (33, 34). This finding may be the result of the committee showing unfailing dedication to fulfilling their members’ interests, increasing the financial risk protection self-assurance of members, which in turn improves their trust and satisfaction with the scheme.

### Strength

This study used a qualitative approach in an attempt to understand why HHs drop out of CBHI.

### Limitation

It was better to match the qualitative participants with different compositional and contextual factors to minimize the confounder. Although this is logical, several assumptions have been made in the Discussion when comparing the findings with previous studies. In addition, desirability bias and recall bias were among the drawbacks; however, there was an attempt to decrease recall bias by reducing the health care utilization time to <3 months for the data collection period.

## Conclusion

This study revealed several variables that affected HHs’ satisfaction with CBHI, including wealth status, HH head’s knowledge of CBHI, illness, familiarity of HHs with medical facilities, graduation from a model household, confidence in the CBHI governing board, ability to obtain prescribed medications from contracted medical facilities, and length of time enrolled in the CBHI program. We strongly advise all responsible parties to focus on increasing HHs’ knowledge of the CBHI scheme, supporting HHs to serve as role models for rural health extension packages, and completing the CBHI pledged package to improve HHs’ satisfaction with the CBHI scheme, which may then play its part in the sustainability of CBHI.

## Data availability statement

The original contributions presented in the study are included in the article/supplementary material, further inquiries can be directed to the corresponding author.

## Ethics statement

The studies involving human participants were reviewed and approved by Ethical review board of Wolkite University. The patients/participants provided their written informed consent to participate in this study.

## Author contributions

The author confirms being the sole contributor of this work and has approved it for publication.

## Funding

There was no external funding for this study. Wolkite University cover some of the budget for data gathering and organization. However, the university did not cover the publication fee.

## Conflict of interest

The author declares that the research was conducted in the absence of any commercial or financial relationships that could be construed as a potential conflict of interest.

## Publisher’s note

All claims expressed in this article are solely those of the authors and do not necessarily represent those of their affiliated organizations, or those of the publisher, the editors and the reviewers. Any product that may be evaluated in this article, or claim that may be made by its manufacturer, is not guaranteed or endorsed by the publisher.
